# Epigenetic effects toward new insights as potential therapeutic target in B-thalassemia

**DOI:** 10.1186/s43141-021-00138-x

**Published:** 2021-03-31

**Authors:** Noha Hamdy Eltaweel, Ghada Youssef ElKamah, Rabab Khairat, Hanan Abd Elmawgoud Atia, Khalda S. Amr

**Affiliations:** 1 Medical Molecular Genetics Department, Human genetics and genome project Division, National Research Centre, El Buhouth St., Dokki, Cairo, 12622 Egypt; 2 Clinical Genetics Department, Human genetics and genome project Division, National Research Centre, Cairo, Egypt; 3 Pharmacology and Toxicology Department, College of Pharmacy, Hail University, Hail, Saudi Arabia; 4 Biochemistry Department, Faculty of pharmacy (Girls), Al-Azhar University, Cairo, Egypt

**Keywords:** Thalassemia therapy, HbF induction, MicroRNA, miR-15a, miR-16-1, miR-96, miR-486-3p

## Abstract

**Background:**

Fetal hemoglobin (HbF) induction has shown promise for the treatment of β-hemoglobinopathies. HbF induction in β-thalassemia could overcome ineffective hematopoiesis and thus terminate transfusion dependency for formerly transfusion dependant patients. Several miRNAs have been found to reactivate γ-globin expression and increase HbF. In this study, we aimed to investigate the expression of 4 miRNAs (miR-15a, miR-16-1, miR-96, and miR-486-3p) in high HbF thalassemia patients and correlate their levels with the patients’ HbF levels then, in order to predict the exact role of the studied miRNAs in hematopoiesis, a bioinformatic analysis was carried out. We went through this bioinformatic analysis to determine the network of genes regulated by miRNAs and further investigate the interaction between all of them through their involvement in hematopoiesis. In this study, the differential expression was measured by qRT-PCR for 40 patients with high HbF and compared to 20 healthy controls. Bioinformatics was conducted involving functional annotation and pathway enrichment analyses.

**Results:**

The studied microRNAs were significantly deregulated in thalassemia patients in correlation with HbF. Functional annotation and pathway enrichment analyses revealed a major role of miR-486-3p and miR-15a in HbF induction.

**Conclusion:**

MiR-486-3p and miR-15a are crucial for HbF induction. Further validating studies are needed.

## Background

Hemoglobinopathies, especially thalassemias are one of the commonest single gene disorders [10]. Thalassemia is considered a global health problem with a high incidence in the Mediterranean populations, Arabs, and Asians [39].

Thalassemia is an autosomal recessive hereditary blood disorder characterized by chronic hemolytic anemia due to improper hemoglobin (Hb) synthesis. Insufficient β-globin production results in excess unpaired α-globin chains that precipitate within erythroid precursors causing premature death of these precursors and ineffective erythropoiesis [46]. Normally, the process of globin chain production keeps the ratio of α-chains to non-α-chains, at 1:1 (± 0.05), but in thalassemia this ratio is disrupted [19]. The clinical severity of β-thalassemia is related to the extent of imbalance between the α- and non-α-chains (β-, δ-, and γ-globin chains) [44]. Inducing HbF production is a novel therapeutic goal for hemoglobinopathies patients. Induced γ-globin chains can balance the pathological α-globin chains, thus, improving anemia and terminating transfusion dependency [20, 24]. Several pharmacological HbF inducers were tested mainly histone deacetylases (HDACs), short-chain fatty acids (SCFAs), and hydroxyurea (HU). Both histone deacetylases and SCFAs are not widely used because of concerns about potential carcinogenicity. HU is the only commercially available drug for HbF induction. It reduces the frequency and severity of sickle cell crises and may inhibit end-organ damage [43]. However, HU has some limitations; it has inconsistent effectiveness (only in 50% of SCD cases), and limited utility for the treatment of β-thalassemia [1]. Therefore, there is an urging demand for new safe and effective alternatives.

Several miRNAs were identified as deeply involved in the erythroid cells, regulating its proliferation, maturation, and expression of fetal globin genes [5]. Both miR-15a and miR-16-1 appear to cause the elevated embryonic and fetal hemoglobin levels in newborns in human trisomy 13 through direct inhibition of MYB which is a potent silencer of fetal and embryonic hemoglobin genes [45]. MiR-486-3p is a direct inhibitor of BCL11A gene expression which in turn control and inhibit γ-globin gene expression [37]. In contrast, miR-96 directly inhibits γ-globin gene expression through the human erythropoiesis [3].

In this study, we aimed to have an overview on the selected miRNAs (miR-15a, miR-16-1, miR-96, and miR-486-3p) as potential therapeutic target. This overview included the differential expression of the four miRNAs in thalassemic patients with high HbF compared to controls. Secondly, investigating the potential role of the studied microRNAs either directly or indirectly in different major mechanisms affecting hematopoiesis and the hemoglobin switching process, using different bioinformatic analysis tools. Up to our knowledge, this is the first study that performed the relative expression of miR-15a, miR-16-1, and miR-486-3p in high HbF thalassemia cases. Studying such miRNAs and their role in normal blood physiology is urgently needed to open the door for new therapies for β-hemoglobinopathies.

## Novelty statement

Our work presents unique study of 4 selected microRNAs crucial for HbF induction first by qRT-PCR then by bioinformatics tools.

The central finding of our work: the major role of miR-486-3p and miR-15a in HbF induction.

The clinical relevance of our work: it could help in introducing novel HbF inducers which could help in treating patients with beta thalassemia and sickle cell disease as well.

## Methods

### Patients

Forty Egyptian beta-thalassemia patients (with high HbF) presented with different degrees of clinical severity were recruited from the Hereditary Blood Disorders Clinic, Center of Medical Excellence, National Research centre (NRC), Egypt over the period from January 2016 to October 2017 from different areas of Upper and Lower Egypt, in addition to 20 sex- and age-matched healthy controls with negative family history of thalassemia or any other anemias. All patients were subjected to clinical evaluation, lab investigations including CBC, serum ferritin, and hemoglobin electrophoresis, in addition to molecular diagnosis. For transfusion-dependent patients, blood samples were collected just before receiving blood transfusion.

### Blood sample collection

Five milliliters of blood samples were collected from 40 thalassemic patients and 20 controls, each in Vacutainer® tubes with EDTA. Plasma was separated within maximum 4 h after blood collection and stored at − 40 °C.

### Total RNA isolation and miRNA quantification

Total RNA was extracted from plasma using miRNeasy mini kit (QIAGEN, CA, USA) according to the manufacturer protocol for plasma/serum samples. Fifty nanograms from each RNA sample were used for reverse transcription reaction. Targeted miRNAs quantification was carried out using the miScript PCR System (QIAGEN, CA, USA) (miScript II RT Kit, miScript SYBR Green PCR Kit (QIAGEN, Valencia, USA), and specific miScript Primer Assays for each miRNA) using comparative method. The results were normalized with SNORD68. Relative changes in gene expression (Rq) were calculated using 2^-∆∆CT^ method [36].

### Statistical methods

Data analyses was conducted using Statistical Package for the Social Sciences (SPSS) software (SPSS Inc., Chicago, USA), version 19.0. Results were expressed as mean (SD). Statistical differences were considered significant at *P* < 0.05. The Shapiro-Wilk test was used to check normality. Student’s *t* test was run to test the significance of the differences in means in the studied variables between the 2 groups. MiRNA expression levels were compared using the non-parametric Mann-Whitney’s *U* test. The Spearman’s rank correlation coefficient (*r*) was calculated.

### MicroRNA target gene prediction

To gain a comprehensive view of the potential target genes of the studied miRNAs, especially they are decided to be used as therapeutic targets, the MiRWalk database V.2.0 (http://zmf.umm.uni-heidelberg.de/apps/zmf/mirwalk2/) [17] was used to identify predicted and validated target genes of each miRNA. We retrieved prospective target genes found in the following 4 online databases simultaneously: miRWalk2.0, RNA22, miRanda, and Targetscan. Genes that were identified in the 4 databases were chosen for further analysis to improve the reliability of the predictions made. In this study, the predicted miRNA-binding sites within the 3′ UTR of genes in the entire human genome were studied.

### Functional enrichment analysis

GO annotation and KEGG pathways enrichment analyses were carried out (based on the selected target genes) using the Database for Annotation, Visualization and Integrated Discovery (DAVID) (https://david.ncifcrf.gov/). Kyoto Encyclopedia of Genes and Genomes (KEGG) was used to perform pathway analysis and to clarify the functional mechanism of the target genes. In GO and KEGG pathway analyses, *q* value (Benjamini) < 0.01 was considered to be highly significantly enriched.

### Network construction and analysis

In order to assess any other possible effect of the studied microRNAs on the globin genes and their interacting proteins, we used the Search Tool for the Retrieval of the Interacting Genes (STRING) available at (https://string-db.org/) to search and construct an interaction network of the different globin genes with other possible target genes of the studied microRNAs [48]. STRING provides both experimental and predicted protein-protein interaction information. All associations are provided with a combined confidence score depending upon prediction (neighborhood in different species, occurrence in the same pathway, and observed co-expression of genes), besides experimentally determined interactions, database annotations, and automated text-mining of scientific texts (e.g., OMIM, PubMed). STRING also imports protein associations from databases of physical interaction and databases of biological pathways (e.g., MINT, HPRD, BIND, BioGRID, KEGG, Reactome, IntAct, GO). Each score represents a rough estimate of how likely a given association describes a functional linkage between two proteins (> 0.5 = medium confidence, > 0.7 = high confidence, > 0.9 = highest confidence). The pairs with a combination score > 0.7 were selected. The whole data were used to construct a network using Cytoscape platform: https://cytoscape.org/.

## Results

### Demographic data and clinical presentation of the studied groups

Patients recruited in our study were referred from all over Egypt (27.3% from Upper Egypt, 40.9% from Nile delta and 31.8% from Greater Cairo). Seventy-five percent of the recruited patients received regular blood transfusion at different intervals, while 20% did not receive any blood transfusion. The remaining 5% were transfused only once. Clinical data of the studied cases are shown in Table 1.

**Table 1:** Clinical data of the studied groups

Groups	Patients (*n* = 40)	Controls (*n* = 20)	*P* value
Variables			
Sex			
Male (*n*-%)	20–50%	14–70%	0.26
Female (*n*-%)	20–50%	6–30%	
Age (years)			
Range	0.5–16	6–12	0.06
Mean ± SD	5.16 ± 4.7	8.4 ± 2.12	
Age of onset (month)			
Range	3-36	–	
Mean ± SD	12.87 ± 10.7		
Interval of transfusion (weeks)			
Range	3–8	–	
Mean ± SD	5.1 ± 2.02		
Hemoglobin (g/dL)			
Mean ± SD	7.2 ± 1.3	12.18 ± 1.0	< 0.0001***
Hematocrit (%)			
Mean ± SD	21.4 ± 4.0	38.9 ± 2.6	< 0.0001***
RBCs count (×10^6^/μL)			
Mean ± SD	3.0 ± 0.44	4.66 ± 0.49	< 0.0001***
MCV (fL)			
Mean ± SD	65.6 ± 12.5	87.29 ± 5.23	< 0.0001***
MCH (pg)			
Mean ± SD	20.8 ± 5.2	29.2 ± 2.37	< 0.0001***
RDW (%)			
Mean ± SD	28.0 ± 8.3	13.3 ± 1.58	0.002**
PLT (×10^6^/μL)			
Mean ± SD	426 ± 36.8	308 ± 54	0.004**
TLC (×10^3^/μL)			
Mean ± SD	16.1 ± 1.9	6.1 ± 1.7	0.001**
Reticulocytes count (%)			
Mean ± SD	4.8 ± 1.28	1.4 ± 0.39	0.02*
Serum Ferritin (ng/ml)			
Mean ± SD	1161.8 ± 245	143.4 ± 24.49	0.003**
Hemoglobin electrophoresis			
HbA (%): Mean ± SD	42.5 ± 30.6	97.55 ± 0.5	< 0.0001***
HbF (%): Mean ± SD	53.5 ± 33.5	0.58 ± 0.18	< 0.0001***
HbA_2_ (%): Mean ± SD	2.8 ± 1.4	1.76 ± 0.56	0.001**

### Relative expression levels of target microRNAs in patients vs controls

**Fig. 1:**
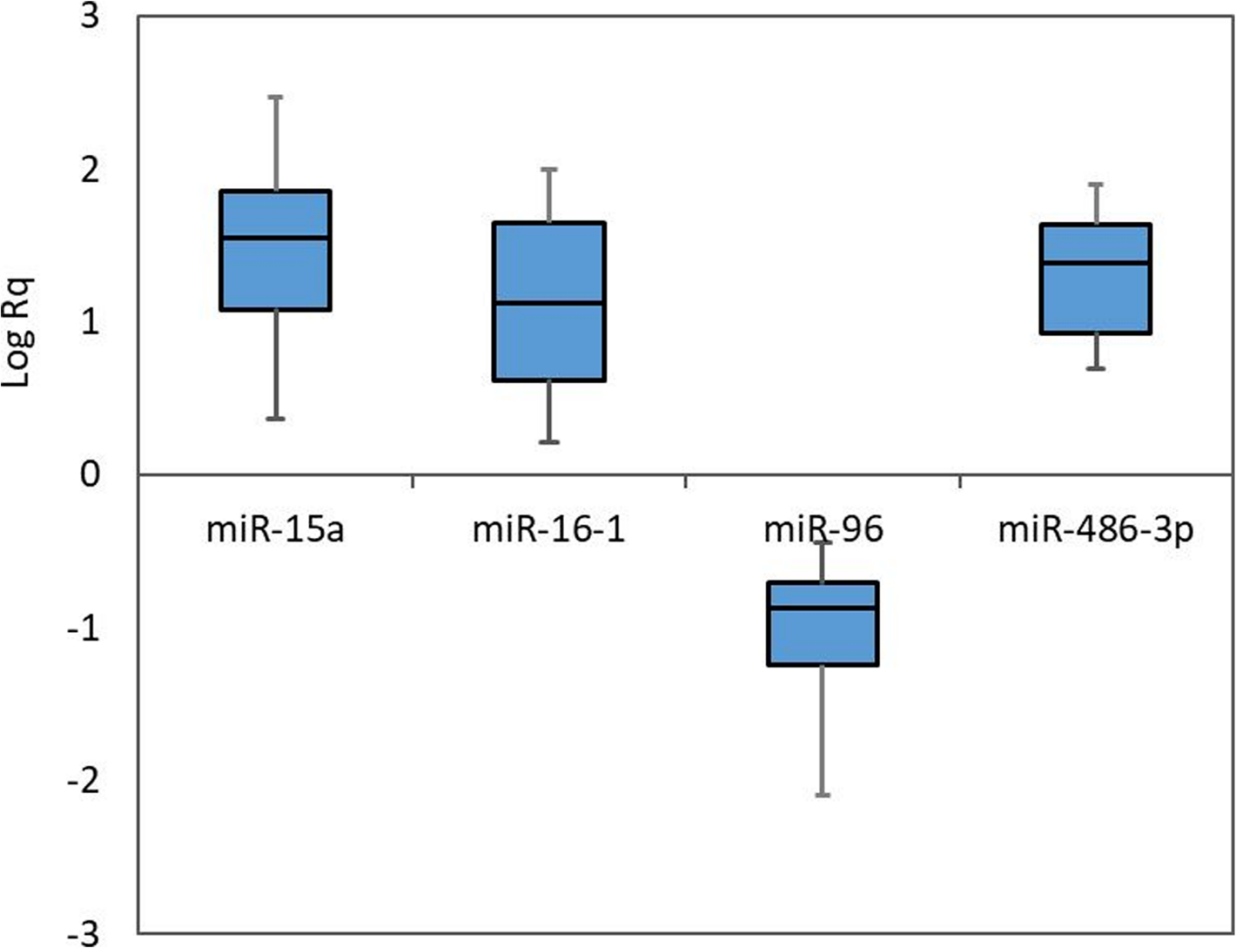
Box and whisker plots representing the expression of the 4 miRNAs in the studied groups (the plot shows the maximum and minimum values, the median, Q1, Q3, and the interquartile range)

Mean (SD) of relative quantification “Rq” of target miRNAs in the studied groups are shown in (Fig. 1). MiR-96 was significantly downregulated (FC = 0.13), while miRNA-15a, miRNA-16-1, and miRNA-486-3p were highly significantly upregulated (FC = 42.97, 17.8, and 21.45, respectively) in patients compared to healthy controls (*P* < 0.0001 for all).

**Fig. 2:**
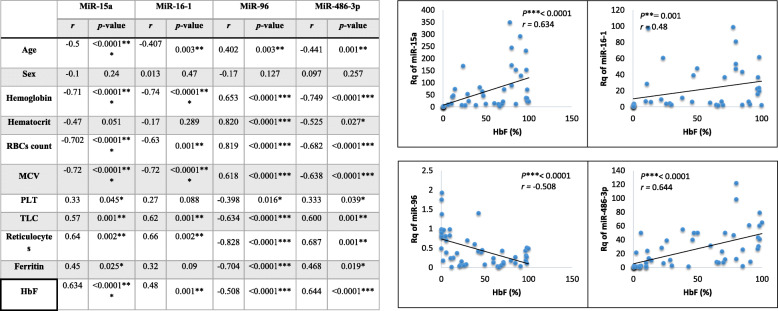
Correlation coefficient between Rq of the studied miRNAs and the clinical data, correlations with HbF is shown in the graph

The correlation analyses of the Rq of the studied miRNAs vs. clinical data of the cases are shown in Fig. 2. MiR-96 expression was found inversely correlated with HbF level with highly significance; on the other hand, a highly significant positive correlation between miR-15a, miR-16-1, and miR-486-3p and HbF was found, as shown in Fig. 2.

### MicroRNA target prediction

The output data obtained though application of 4 mentioned online prediction databases revealed that 2642 genes that appeared in them all simultaneously were subsequently considered as predicted target genes for the upregulated miRNAs (miR-15a, miR-16-1, miR-486-3p), and 550 genes for the down-regulated miRNA (miR-96).

### Enrichment analysis and bioinformatics of target genes

**Fig. 3:**
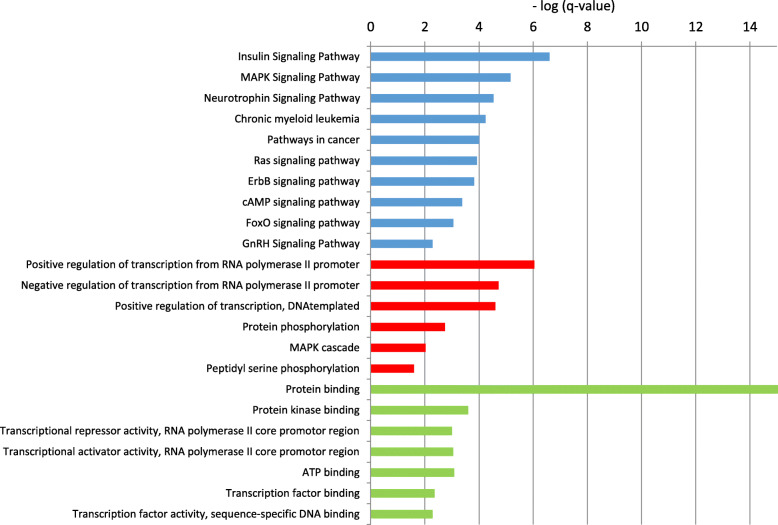
The GO and KEGG pathway analyses of the upregulated microRNAs. Blue bars represent highly enriched KEGG pathways, while red and green bars represent highly enriched biological processes and molecular functions, respectively. *q* value: FDR adjusted *p* value

The pathway enrichment analysis (blue bars) revealed that 25 pathways were highly enriched for the target genes of the upregulated miRNAs studied. Among them, the highly enriched pathways related to hematopoiesis were MAPK [21], insulin signaling pathway [2, 40], neurotrophin signaling pathway [18], Ras [27], FOXO [38], and mTOR signaling pathways [50], in addition to pathways involved in cancer especially AML and CML as shown in Fig. 3.

Through GO enrichment analysis, biological process (BP) and molecular function (MF) were highlighted. In the biological process module (red bars), 10 terms were significantly enriched and many of them were related to regulation of transcription of what genes. In molecular function domains (green bars), 10 terms were significantly highly enriched, the analysis mainly noted protein binding, transcriptional repressor and activator activity, ATP and transcription factor binding.

**Fig. 4:**
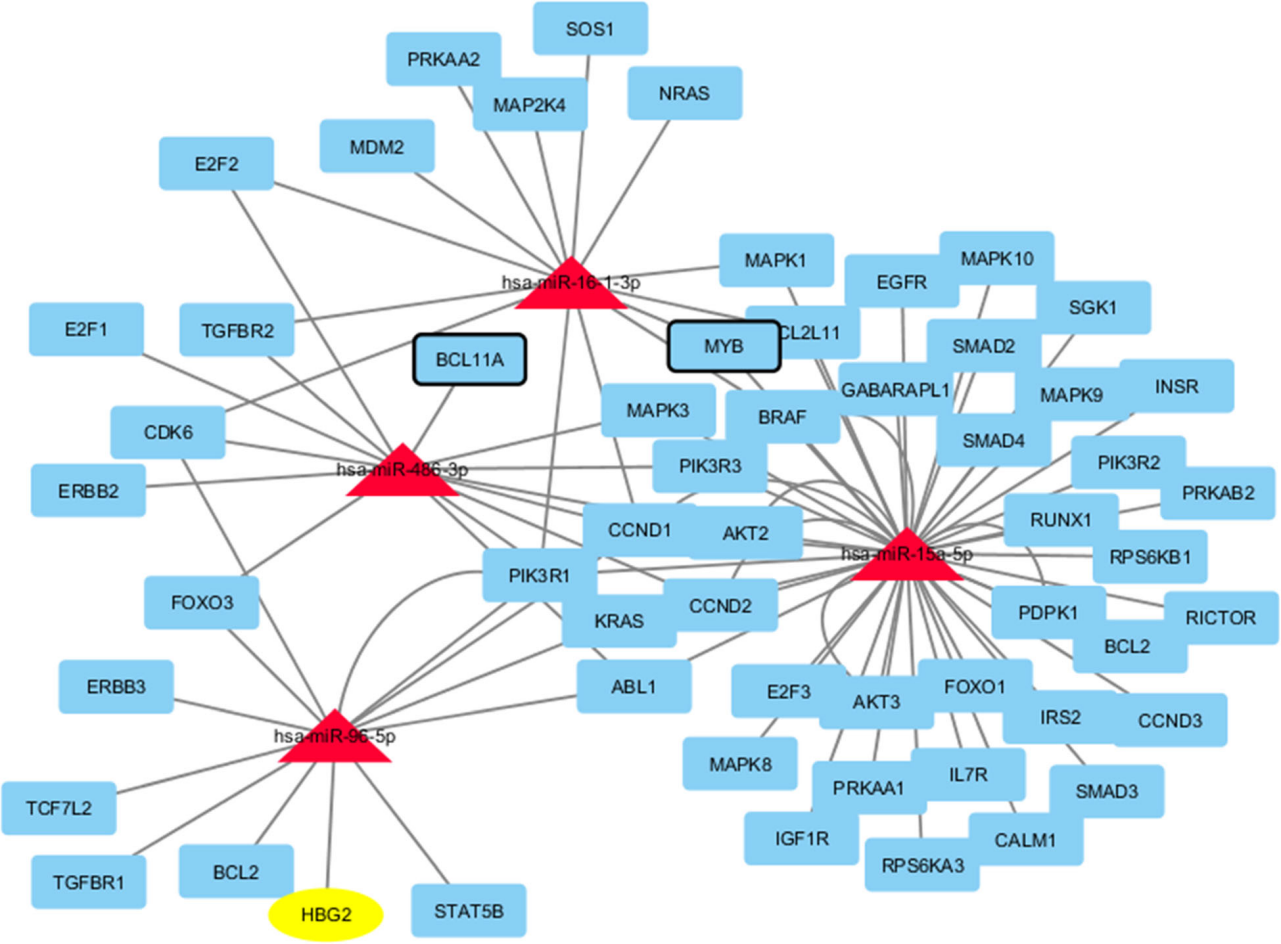
The studied microRNAs and their target genes involved in highly enriched pathways related to hematopoiesis

The target genes involved in the highly enriched pathways and GO terms are represented in the following network drawn by Cytoscape (Fig. 4).

### The potential role of the studied miRNAs in the whole hematopoiesis process

The possible role of each microRNA in hematopoiesis was predicted through reviewing the role of their putative target genes in the literature. The results are shown in Table 3.

### The indirect interaction of the studied miRNAs with globin genes

**Fig. 5:**
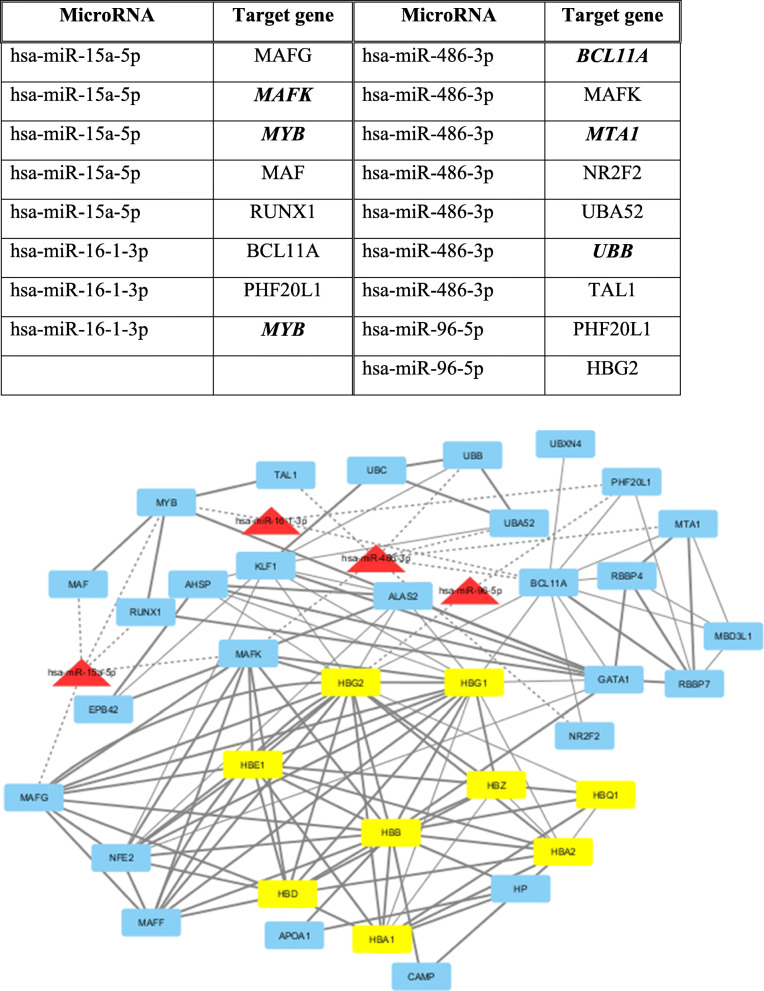
Network of the globin proteins and their interacting proteins, besides the possible link with the studied microRNAs, drawn by cytoscape. The red triangles are the microRNAs, the yellow ovals represent the globin proteins, while the blue round rectangles are other proteins. The width of the edge represents the confidence score where the bold solid lines are > 0.9 while normal solid are > 0.7. MicroRNA interactions are represented by dashed lines. Bold genes in the table are those validated as targets of the corresponding miRNA

Using the STRING database, the protein-protein interactions involving the globin genes were exported. The major interacting proteins with the globin genes with the highest score (> 0.9) were HP, MYB, GATA1, NFE2, CAMP, APOA1, MAFG, MAFK, and MAFF. The interacting genes targeted (either validated or predicted) with our studied microRNAs are shown in Fig. 5.

## Discussion

The study of hemoglobin switching process represented a focus in hematology to ameliorate the severity of β-hemoglobinopathies. Additionally, the process by which this switch occurs represents an important paradigm for developmental gene regulation and the possible interaction of different microRNAs in this process. In the current study, the relative expression of four microRNAs (mir-96, mir-486-3p, mir-15a, and mir-16-1) in high HbF Egyptian thalassemia patients were found to be differentially expressed compared to controls and could also predicted that had a potential role on hematopoiesis and hemoglobin switching processes.

### Relative expression levels of target microRNAs in patients vs. controls

Our study revealed that the Egyptian thalassemia patients had concordant miR-96 profile with the previous records in other populations, such that miR-96 expression in high HbF cases was about 10-fold lower than controls. On the other hand, miR-15a, miR-16-1, and miR-486-3p expression in high-HbF cases were showed significantly high expression than controls (FC = 42.97, 17.8, and 21.45, respectively; Fig. 1).

Moreover, miR-96 expression levels were found inversely correlated with HbF (Fig. 2), in accordance with Azzouzi et al. [3]. In the same context, Noh et al. [42] stated that miR-96 was upregulated and the most differentially expressed on the arrays with 10-fold increase in adult blood versus cord blood when they examined the erythrocyte microRNA profiles in adults and at birth to correlate with hemoglobin switching phenomenon.

On the other hand, a highly significant positive correlation between miR-486-3p and HbF was found (Fig. 2) in agreement with Lai et al. [31]. Lulli et al. [37] showed that miR-486-3p is a critical inducer of HbF level, through direct inhibition of BCL11A gene expression. Moreover, BCL11A is a major regulator of HbF switching and critical for the maintenance of γ-globin gene silencing in adult human erythroid cells. The study was carried out on erythroid cells of patients affected by major or intermedia β-thalassemia, and they assumed that the observed variable miR-486-3p expression levels among β-thalassemic patients might be the cause of the observed different HbF content among those patients and possibly to the clinical severity of the disease.

It is worse to mention that Hojjati et al. [26] examined in vivo expression of some miRNAs thought to be involved in pharmacological induction of HbF among β-thalassemia patients who were undergoing hydroxyurea (HU) therapy. Expression of γ-globin and miR-486-3p had higher levels in responders than non-responders group, but the expression of miR-96 did not show any significant difference between the study groups. These results highlight the importance of mir-486-3p in the mechanism of HPF switching process.

Also, our results showed that miR-96 had positive correlation with age (*r* = 0.402, *P* = 0.003). This positive correlation was reported also by Budzinska et al. [8] where the expression of miR-96 increases upon aging, while miR-486-3p showed negative correlation with age (*r* = − 0.441, *P* = 0.001). This finding meet the study of Lai et al. [30] who found that miR-486 has age-related expression such that it is constantly expressed in infancy and childhood, then downregulated in young adulthood, and then diminished with aging.

Additionally, expression levels of miR-15a and miR-16-1 were found to be correlated with the level of HbF (Fig. 2); these results go with Sankaran and coworkers [45] who reported that elevated HbF expression in human trisomy13 is due to increased expression of miR-15a and miR-16-1.

### Role of the studied microRNAs in hematopoiesis

We conducted a computational analysis to examine the effect of the studied microRNAs on hematopoiesis in general. We found a link between the studied microRNAs with both the whole hematopoiesis process, as well as their indirect interaction with globin genes through intermediate genes. The enrichment analysis revealed the highly enriched pathways related to hematopoiesis as shown in Fig. 3. MAPK signaling pathways are essential in the regulation of multiple processes involved in blood cell production, in regulation of hematopoiesis in general and myelopoiesis in particular and also it has been demonstrated to play a key role in the maintenance of HSC (Hematopoietic stem cells) quiescence. Moreover, MAPK pathways play critical role in the pathogenesis of various hematological malignancies such that aberrant activation of this pathway could be considered as a likely cause of hematopoietic disease [21].

Many signaling molecules affect hematopoiesis either directly or indirectly. Of them, insulin growth factors (IGF-1 and IGF-2) and insulin signaling pathway have a major function in hematopoiesis. IGF-1 stimulates erythropoiesis in vitro and in vivo [2, 40] as well as the neurotrophin signaling pathway [18]. Moreover, Ras is one of the signaling molecules activated during erythropoiesis and appear important for the balance of proliferation/differentiation/apoptosis of erythroid cells [27]. Also, FoxOs genes known to regulate intracellular reactive oxygen species (ROS) levels such that loss FoxOs results in elevated ROS levels, which in turn negatively regulate cellular responses to erythroid differentiation. It was reported that FoxOs including FoxO3a regulate hematopoietic stem cell function by controlling oxidative stress and cell cycling. FoxO3a plays a pivotal role in maintenance, integrity, and stress resistance of HSCs through negative feedback pathways. It also regulates hematopoietic homeostasis [38]. Another signaling molecule is mTOR protein kinase that responds to multiple signals and maintains homeostasis during embryonic development and adulthood. Altered mTOR activity can alter HSC function and cause hematological disorders [50].

The target genes either validated or predicted involved in the enriched pathways are shown in Table 2. It was demonstrated that AKT3 is a direct target of miR-15a and miR-16 and both AKT2 and AKT3 are critical regulators of HSC function [28, 47]. Both miR-15 and miR-16 are considered as tumor suppressors that could be used for therapy of inhibiting BCL2-, CCND1-, and CCND2-overexpressing tumors, e.g., chronic lymphocytic leukemia, breast cancer, prostate, and lung cancer [4, 7, 9, 11, 12]. Moreover, miR-96 is known as a tumor suppressor in pancreatic cancer by suppressing KRAS [29], as well as in endometrial and breast cancers [23, 34, 41] by suppressing FoxO1 and FOXO3 genes. FoxO1 gene is also a validated target for miR-15a [15].

**Table 2:** Enriched pathways and the involved target genes of the studied miRNAs in each pathway

Pathway	Target genes of the studied microRNAs involved in that pathway	
	Predicted	Validated
Insulin signaling pathway	AKT2, BRAF, KRAS, SOS1, PDPK1, CALM1, ERS2, INSR, MAPK1, MAPK3, MAPK8, MAPK9, MAPK10, NRAS, PIK3R1, PIK3R2, PIK3R3, PRKAA1, PRKAA2, PRKAB2, RPS6KB1	AKT3, KRAS, FOXO1
MAPK signaling pathway	AKT2, BRAF, KRAS, SOS1, EGFR, MAPK1, MAPK3, MAPK8, MAPK9, MAPK10, MAP2K4, NRAS, TGFBR1, TGFBR2	AKT3, KRAS, RPS6KA3
Neurotrophin signaling pathway	PDPK1, ABL1, AKT2, BRAF, KRAS, SOS1, CALM1, BCL2, FOXO3, PIK3R1, MAPK1, MAPK3, MAPK8, MAPK9, MAPK10, NRAS, PIK3R1, PIK3R2, PIK3R3,	AKT3, KRAS, BCL2, FOXO3, RPS6KA3
Chronic myeloid leukemia	ABL1, AKT2, E2F1, E2F2, E2F3, BRAF, KRAS, MDM2, SMAD4, SOS1, CCND1, CDK6, MAPK1, MAPK3, NRAS, PIK3R1, PIK3R2, PIK3R3, RUNX1, STAT5B, TGFBR1, TGFBR2	AKT3, KRAS, CCND1
Ras signaling pathway	ABL1, AKT2, KRAS, SOS1, CALM1, IGF1R, EGFR, INSR, MAPK1, MAPK3, MAPK8, MAPK9, MAPK10, NRAS, PIK3R1, PIK3R2, PIK3R3	AKT3, KRAS
FOXO signaling pathway	PDPK1, AKT2, BRAF, KRAS, SOS1, BC2L11, GABARAPL1, MDM2, SMAD2, SMAD3, SMAD4, CCND1, CCND2, EGFR, FOXO1, FOXO3, IGF1R, IRS2, INSR, IL7R, MAPK1, MAPK3, MAPK8, MAPK9, MAPK10, NRAS, PIK3R1, PIK3R2, PIK3R3, PRKAA1, PRKAA2, PRKAB2, SGK1, TGFBR1, TGFBR2	AKT3, KRAS, CCND1, CCND2
GnRH signaling pathway	KRAS, SOS1, CALM1, EGFR, MAPK1, MAPK3, MAPK8, MAPK9, MAPK10, MAP2K4, NRAS	KRAS

The possible role of each microRNA in hematopoiesis was predicted through reviewing the role of their putative target genes in the literature. Studies involved silencing of one of these target genes using miRNAs were extracted from the literature, and tabulated. The results are shown in (Table 3).

**Table 3:** The expected role of the miRNAs in hematopoiesis through their putative target genes (bold miR are validated)

miRNA	Target gene	Predicted role in hematopoiesis	Reference
miR-486-3p	E2F1	Inhibits granulocytic proliferation and activity	[25]
**miR-15a** **miR-16-1**	cMyb	• Drives MK differentiation • Block DN3 to DN4 T-cell transition • promotes differentiation of bi-potent K562 cells into MKs • the forced expression of miR-15a in bone marrow mononuclear cells blocked the erythroid transition from BFU (erythroid burst-forming units) to CFU (erythroid colony-forming units)	[25, 32]
**miR-15a** **miR-16-1** **miR-96**	BCL2	• Modulate T cell development • Regulation of positive selection by governing the homeostasis	[25]
**miR-15a**	RUNX1	Highly expressed in megakaryocytopoiesis	[33]
**miR-486-3p** **miR-96**	FOXO3	Deprotect erythroid cells from oxidative stress	[25]
miR-96 **miR-16-1**	CDK6	Prognostic marker for Mantle cell lymphoma	[49]
**miR-15a** **miR-16-1** miR-486-3p	CCND1	Protect against Mantle cell lymphoma	[49]
miR-96 **miR-15a**	ABL1	Involved in CML	[35]
miR-96 miR-15a	KRAS	Tumor suppressors	[29]

Kras has an important function in adult hematopoiesis and its loss might develop profound hematopoietic defects and is prone to myeloid diseases [14]. On the other hand, the abnormal activation of Kras had been found to contribute to Kras-driven tumorigenesis. So, miRNAs that target and regulate Kras could have tumor-suppressive role [29].

More studies are needed to confirm the effect of the studied microRNAs (miR-15a, miR-16-1, miR-96, and miR-486-3p) on these suggested pathways to answer the puzzle of their role.

### Indirect interaction of the studied microRNAs with globin genes

From protein-protein interaction study using STRING, many interacting genes with the globin genes are putative targets of the studied microRNAs as shown in Fig. 5. Globin gene expression is regulated through nuclear factor erythroid-2 (NFE2) elements located in enhancer-like locus control regions positioned many kb upstream of α- and β-gene clusters. NFE2 DNA-binding activity consists of a heterodimer containing a small MAF protein (MAFF, MAFG, or MAFK) [6]. MAF proteins are essential for activation of β-globin gene expression [16]. Our target prediction revealed that MAFG is a target for miR-15a while MAFK is a target for miR-15a and miR-486-3p. Therefore, both miR-15a and miR-486-3p are β-globin gene silencers.

Moreover, NR2F2 is thought to play an important role in the stage-specific silencing of the ε- and γ-globin genes [13]. On the same context, MTA1 is a component of the methyl binding domain 2 (MBD2)-nucleosome remodeling and deacetylating (NURD) complex. MBD2-NURD complex contributes to DNA methylation-dependent embryonic and fetal β-globin gene silencing during development [22]. Both NR2F2 and MTA1 are target genes for miR-486-3p. Together with the previously reported study by Lulli et al. [37] which revealed that BCL11A (γ-globin silencer) is a target of miR-486-3p, these results suggest that miR-486-3p is a potent γ-globin enhancer via different targets.

Moreover, from the protein–protein interaction, a putative target of miR-486-3p is TAL1 gene which is considered as a key of hematopoietic transcription factor that binds to regulatory regions of a large cohort of erythroid genes. In addition, TAL1 plays an important role for γ-globin expression [51].

The correlation of the studied micoRNAs with the predicated genes was not reported before and further wet lab studies are needed to confirm this correlation.

## Conclusions

The studied microRNAs have a potential role in hematopoiesis through affecting other genes mediating signaling pathways or interacting with globin genes. MiR-486-3p overexpression is thought to suppress β-globin gene by targeting MAFK gene, and induce γ-globin gene expression by targeting BCL11A, MTA1, and NR2F2 leading to HbF overexpression. In the same context, miR-15a overexpression is thought to favor hemoglobin switching toward increasing HbF expression by targeting several MAF proteins and MYB gene. Our results need to be more validated via wet lab methods, and that is what we are going to do soon.

## Data Availability

Raw data supporting the findings of this study are available from the corresponding author [Khalda S. Amr] upon request.

## Abbreviations

AKT: Protein kinase B
BCL11A: BAF chromatin remodeling complex subunit
BP: Biological processes
CCND1: Cyclin D1
Ct: Threshold cycle
DAVID: Database for Annotation Visualization and Integrated Discovery
FC: Fold change
FOXO3: Forkhead box O3
GO: Gene ontology
HbF: Fetal hemoglobin
HSC: Hematopoiteic stem cells
KEGG: Encyclopedia of genes and genomes
MAPK: Mitogen-activated protein kinase
MBD2: Methyl binding domain 2
MF: Molecular functions
MTA: Metastasis-associated protein
NFE2: Nuclear factor erythroid-2
NR2F2: Nuclear receptor subfamily 2 group F member 2
NURD: Nucleosome remodeling and deacetylating
Rq: Relative quantitation
SCD: Sickle cell disease
SNORD: C/D box snoRNAs
STRING: Search Tool for the Retrieval of the Interacting Genes
TAL1: T cell acute leukemia 1
UTR: Untranslated region
